# New Species of *Chloroidium* (Trebouxiophyceae, Chlorophyta) from East Asia

**DOI:** 10.3390/plants10122560

**Published:** 2021-11-23

**Authors:** Andrey A. Gontcharov, Arthur Yu. Nikulin, Vyacheslav Yu. Nikulin, Veronika B. Bagmet, Rezeda Z. Allaguvatova, Shamil R. Abdullin

**Affiliations:** Federal Scientific Center of the East Asia Terrestrial Biodiversity, Far Eastern Branch of the Russian Academy of Sciences, 159, 100-Letia Vladivostoka Prospect, Vladivostok 690022, Russia; artyrozz@mail.ru (A.Y.N.); nikulinvyacheslav@gmail.com (V.Y.N.); chara1989@yandex.ru (V.B.B.); allaguvatova@yandex.ru (R.Z.A.); crplant@mail.ru (S.R.A.)

**Keywords:** coccoid green algae, Watanabeales, new species, SSU and ITS rDNA, secondary structure, morphological characteristics, life cycle, temperate monsoon climate zone

## Abstract

*Chlorella*-like green algae that reproduce only asexually by immotile autospores or motile zoospores are the most typical inhabitants of non-aquatic environments. They have a simple morphology that hampers their differentiation, but algae of such habit represent a diverse array of lineages, which are mostly in the classes Chlorophyceae and Trebouxiophyceae. One of these lineages is the order Watanabeales (*Watanabea* clade; Trebouxiophyceae), which comprises 10 genera that share a distinct mode of reproduction through unequally sized autospores. Most of these genera account for a few species that are rarely recorded in nature. In contrast, the genus *Chloroidium* is one of the most species-rich and widely distributed members of the order. Three strains of coccoid green alga were isolated during a study of soil algae in the temperate monsoon climate zone of Asia. These strains are described here as a new species, *Chloroidium orientalis*. SSU and ITS rDNA sequence data, morphological characteristics, and life cycle features differentiate these strains from known members of the genus.

## 1. Introduction

Coccoid spherical and ellipsoid (*Chlorella*-like) green algae reproduce only asexually by immotile autospores or motile zoospores. These organisms are frequent and abundant inhabitants of terrestrial and aquatic (mostly freshwater) environments. This morphotype is typical of many photobionts of lichens as well. They have a simple morphology that hampers taxonomic differentiation, but algae with such a habit represent a diverse array of lineages belonging to the classes Chlorophyceae and Trebouxiophyceae according to the molecular phylogenetic data [1,2,3,4,5,6]. One of these lineages is the *Watanabea* clade in the Trebouxiophyceae, which predominantly comprises terrestrial microalgae that are currently classified in 10 genera. Most of them (seven of the 10) were described based on mainly molecular phylogenetic data and include only a few species with scarce records from nature. Morphological identification of genera and infrageneric taxa comprising *Watanabea* is difficult and could only be achieved with the establishment of their exact molecular phylogenetic affiliation.

Recently, the *Watanabea* clade was defined as the order Watanabeales based on the results of molecular phylogenetic analyses and shared morphological features, especially of unequally sized autospores [7]. The Watanabeales include the genera *Calidiella* Darienko and Pröschold, *Chloroidium* Nadson, *Jaagichlorella* Reisigl, *Kalinella* J. Neustupa, Y. Nemcova, M. Eliás and P. Skaloud, *Massjukichlorella* Darienko and Pröschold, *Mysteriochloris* H.Y. Song, Y.X. Hu, H. Zhu, Q.H. Wang, G.X. Liu and Z.Y. Hu, *Polulichloris* H.Y. Song, Q. Zhang, G.X. Liu and Z.Y. Hu, *Phyllosiphon* J.G. Kühn, *Viridiella* P. Albertano, A. Pollio and R. Taddei, and *Watanabea* N. Hanagata, I. Karube, M. Chihara and P.C. Silva [7]. Among the genera in this order, *Chloroidium* is one of the most species rich (9 spp.) and widely distributed across different non-aquatic habitats. The genus is characterized by narrowly or broadly ellipsoidal to spherical cells, parietal (not cup-shaped) chloroplasts, reproduction by unequally sized autospores in small and large autosporangia as well as by the capability to produce and accumulate sugar alcohol ribitol [8]. *Chloroidium* mostly comprises species that were previously assigned to the genera *Chlorella* Beyerinck [Beijerinck] (*C. ellipsoideum* (Gerneck) Darienko, Gustavs, Mudimu, Menendez, Schumann, Karsten, Friedl and Pröschold, *C. lichenum* (Chodat) Darienko and Pröschold, *C. saccharophilum* (W. Krüger) Darienko, Gustavs, Mudimu, Menendez, Schumann, Karsten, Friedl and T. Pröschold, and *C. viscosum* (Chodat) Darienko and Pröschold), *Parachloroidium* Neustupa and Skaloud (*C. laureanum* (Neustupa and Škaloud) Darienko and Pröschold and *C. lobatum* (Neustupa and Škaloud) Darienko and Pröschold), and *Chlorocloster* Pascher (*C. engadinensis* (Vischer) T. Darienko, Gustavs, Mudimu, Menendez, Schumann, Karsten, Friedl and Pröschold) [8,9]. Recent surveys revealed cryptic species and habitat diversity in Watanabeales [7,10,11,12] and suggested that its species richness is underestimated.

During a study of soil algae diversity in the temperate monsoon climate zone in Russia (Primorsky Territory) and China (Jilin Province), we isolated three strains of *Chlorella*-like coccoid green alga and examined them using an integrative approach. SSU and ITS rDNA sequence data, morphological and morphometric characteristics, and life cycle features differentiate these strains from known members of the genus *Chloroidium* and led us to describe them as a new species, *Chloroidium orientalis*.

## 2. Results

We examined three algal strains isolated from soil (Primorsky Territory, Russia) and concrete wall biofouling (Jilin Province, China) using both phenotypic features and molecular markers. The phylogenetic analysis of concatenated SSU rDNA and ITS rDNA revealed that these strains represent an undescribed *Chloroidium* taxon. Hereafter, we refer to these strains as a new species, *Chloroidium orientalis*.

### 2.1. Taxonomic Treatment

**Figure 1:**
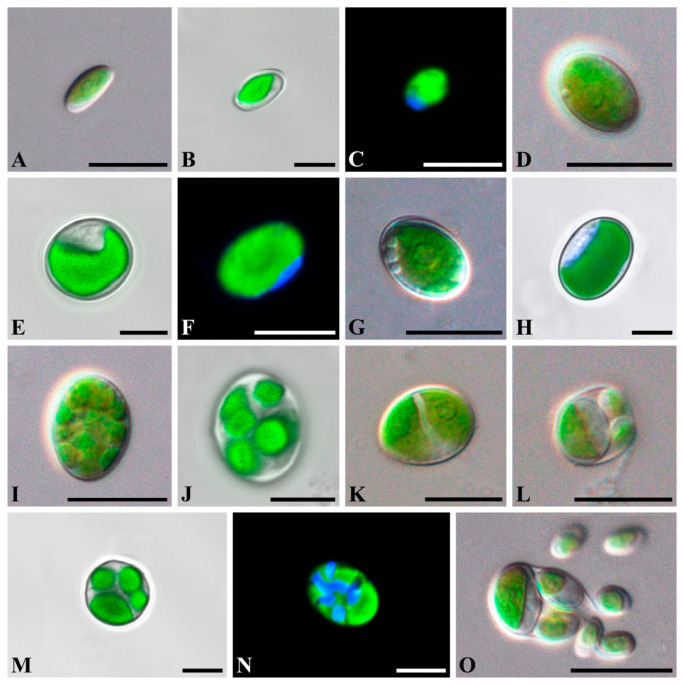
Light micrographs of general morphology (**A**,**D**,**G**,**I**,**K**,**L**,**O**) and confocal reconstructions of chloroplasts and nucleus morphology (**B**,**C**,**E**,**F**,**H**,**J**,**M**,**N**) with bright-field image merged fluorescence channel (**B**,**E**,**H**,**J**,**M**) in *Chloroidium orientalis*. (**A**–**C**) young cell; (**D**–**F**) mature cell; (**G**,**H**) cell with vacuoles; (**I**,**J**) autosporangia with equal small size autospores; (**K**) autosporangia with equal large size autospores; (**L**–**N**) autosporangium with unequal autospores; (**O**) the liberation of the autospores. Scale bars: (**A**,**D**,**G**,**I**,**K**,**L**,**O**) = 10 µm; (**B**,**C**,**E**,**F**,**H**,**J**,**M**,**N**) = 5 µm. (**L**)—strain VLA-CA-1108, others—strain VLA-CA-0963.

**Figure 2:**
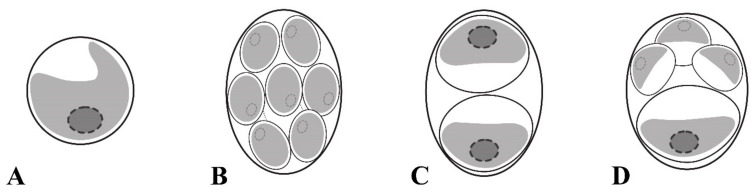
Schematic representation of a mature vegetative cell of *Chloroidium orientalis* having parietal cup-shaped chloroplast with a smooth margin in (**A**), autospores of equal small (**B**), equal large (**C**), and unequal (**D**) size.

*Chloroidium orientalis* Gontcharov, Abdullin, A. Nikulin, V. Nikulin and Bagmet, sp. nov. Figure 1A–O and Figure 2A–D.

Diagnosis: Young cells ellipsoidal to broadly ellipsoidal, 4.1 × 1.8 μm–7.4 × 5.1 μm. The cell wall thin. The chloroplast parietal and band-shaped with a smooth margin. Mature vegetative cells ellipsoidal or broadly ellipsoidal (3.8 × 2.1 μm–16.7 × 9.0 μm) to almost spherical (5.4–10.8 μm in diameter). The chloroplast parietal, band-shaped to cup-shaped, sometimes detached from the cell wall by small vacuoles in the aged cells (Figure 2A). The pyrenoid single, distinct, and covered with several starch grains. The nucleus single, not visible by light microscopy. Reproduction by autospores of equal and unequal size. Autospores of equal small size are produced at 2–16 per sporangium and have a narrowly ellipsoidal shape, 2.1 × 1.5–9.0 × 6.9 μm (Figure 2B). Autospores of equal large size are produced at 2–4 per sporangium and have a broadly ellipsoidal cell-shape, 6.4 × 3.6–10.3 × 8.2 μm (Figure 2C). Autospores of unequal size are produced 4–8 per sporangium: one large broadly ellipsoidal and 3–7 small narrowly ellipsoidal cells (Figure 2D). Autosporangia ellipsoidal or broadly ellipsoidal (7.2 × 5.6–15.4 × 12.1 μm) to spherical (5.6–11.5 μm in diameter). Autospores liberated by apical rupture of the sporangum. The remnants of the sporangial cell wall usually bag-shaped.

Differs from other *Chloroidium* species by the following set of morphological characters: a distinct pyrenoid with several starch grains; autosporangia with three types of autospore sizes (equal small, equal large, and unequal small and large). Also, distinct genetically by differences in the ITS2 sequence and hCBCs in the conservative part of ITS2.

Type locality: Vladivostok, Primorsky Territory, Russia (43°12′44″ N, 131°58′36″ E), forest soil.

Etymology: The species epithet “orientalis” is based on the name of the geographic region (East Asia), where it was found.

Holotype: Exsiccatum number VLA-CA-0963, a dried biomass of unialgal population was deposited in the Herbarium, Federal Scientific Center of East Asian Terrestrial Biodiversity, Vladivostok, Russia. Gene sequence: DNA sequences obtained from clonal strains of *Chloroidium orientalis* were deposited in GenBank under accession no. MZ558750–MZ558752.

### 2.2. Phylogenetic Analyses

The 18S rRNA gene sequence divergence between all *Chloroidium* species was low and did not exceed 1–2% (Table S1). The divergence was sufficiently higher for the ITS1–5.8S–ITS2 region (5–29%; Table 1). ITS sequences in the strains differed from those in other species for more than 11%, while the difference between *C. ellipsoideum* and *C. lichenum* and *C. engadinensis* and *C. viscosum* was sufficiently low, 5.05 ± 0.67% and 8.34 ± 0.96%, respectively. The results of the sequence comparisons suggested that we were likely dealing with a new species. To test this hypothesis, we accessed the phylogenetic position of our strains in the genus *Chloroidium* using a dataset based on analyses of Darienko et al. [9] (Table S2).

**Figure 3:**
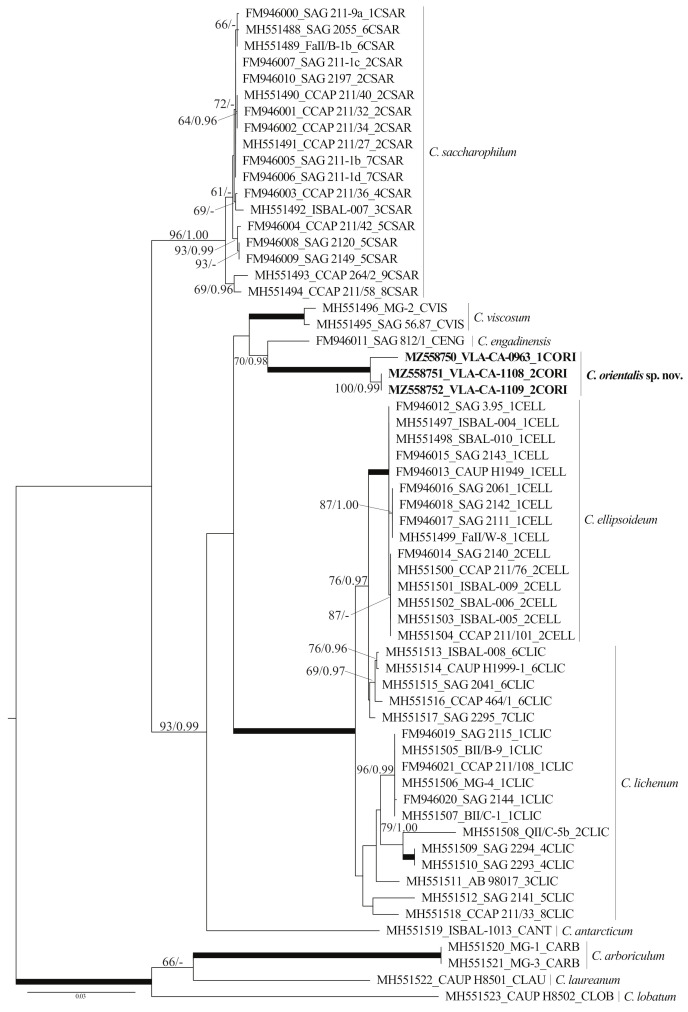
ML phylogenetic tree (GTR + I + G model) showing position of the new species *C. orientalis* (boldface) in the genus *Chloroidium* based on SSU and ITS rDNA sequence data (2471 aligned positions of 61 sequences, 10 species). Support [(BP) ≥ 50% and (PP) ≥ 0.95: ML/BI] are given above/below the branches. Branches with 100% BP, 1.00 PP are shown in boldface. ITS2 ribotypes and clade designations follow Darienko et al. [9].

The dataset included 61 *Chloroidium* accessions representing 9 described species and our isolates. It was 2471 nt long, and of these 332 characters were parsimony-informative. The overall topologies of our unrooted ML and BI trees were similar to that presented by Darienko et al. [9] (Figure 3). For all but the species *C. lichenum*, represented by more than one sequence, respective generic clades were resolved, *C. sacharophyllum*, *C. viscosum*, *C ellipsoideum*, and *C. arboriculum* Darienko and Pröschold. Our strains formed a highly supported (100/1.00) clade that was resolved as a sister lineage to *C. engadinensis* with moderate support (70/0.98). This lineage was a part of the moderately supported clade (93/0.99) formed by *C. antarcticum* Darienko, Lukešová and Pröschold, the *C. ellipsoideum*/*C. lichenum* subclade, and *C. viscosum*. The relationship between these entities remained largely unresolved. One more *Chloroidium* intrageneric clade united long-branched *C. arboricolum*, *C. laureanum*, and *C. lobatum* (Figure 3).

### 2.3. ITS2 Secondary Structure

**Figure 4:**
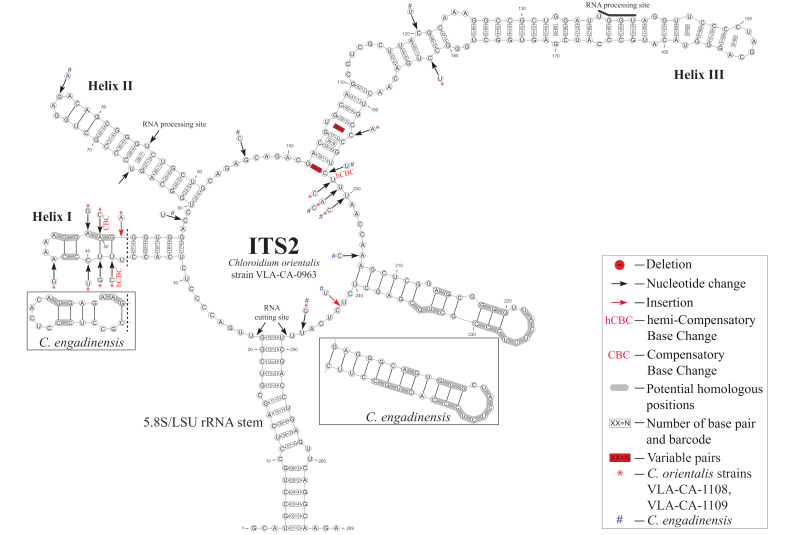
Secondary structure models of ITS2 of *C. orientalis* strains and of *C. engadinensis* based on Mfold predictions. The numbers in frames indicate numeration of each conservative pair used for barcodes and its code: 1 = A-U; 2 = U-A; 3 = G-C; 4 = C-G; 5 = G•U; 6 = U•G; 7 = mismatch; 8 = deletion, single or unpaired bases. See the legend for details.

To analyze relationships between the new species and its sister taxon, *C. engadinensis* (Figure 3), we compared their ITS2 secondary structures. Figure 4 illustrates the proposed base pairing in ITS2 of *C. orientalis* strains and *C. engadinensis*. The spacer had a four-helical domain structure with five single-stranded regions, which is typical for most eukaryotes. Approximately 82% of the nucleotides were involved in the formation of these hairpin loops. The terminal part of helix I and all of helix IV were the most variable domains in these species. Due to this variability, it was somewhat difficult to access homology in these regions between *C. orientalis* and *C. engadinensis* models (Figure 4). We analyzed ITS2 secondary structures in our strains to detect CBC/hCBC events that may mark speciation and found an hCBC in the basal part of the helix III that differentiated *C. orientalis* from *C. engadinensis* (G•U > G-C, 102–197 pair (position 33 in the barcode alignment, see below). In addition, our strains also differed in an hCBC and one CBC in more-variable helix I (G•U > G-C, 38–51 pair, and A-T > G-C, 39–50 pair, respectively), which were not included in the list of conservative ITS2 barcoding positions.

**Figure 5:**
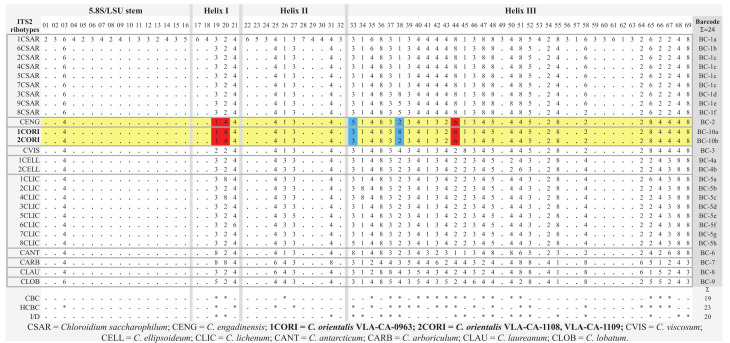
Comparison of the conserved region of ITS2 among the species of *Chloroidium*. Number code for each base pair: 1 = A-U; 2 = U-A; 3 = G-C; 4 = C-G; 5 = G•U; 6 = U•G; 7 = mismatch; 8 = deletion, single or unpaired bases. Compared strains are marked in yellow. Unique CBCs and hCBCs discussed in the text are highlighted. Ribotypes and barcodes revealed in this study are boldfaced. An asterisk marks presence of CBC, hCBC, and indel/deletion.

The conserved regions of ITS2 were transformed into a unique numerical code using the ITS2/CBC approach described by Darienko et al. [9,13] (Figure 4 and Figure 5). *C. orientalis* added two ITS2 ribotypes (1CORI and 2CORI) and two unique barcodes (BC-10a and BC-10b) in addition to 25 ribotypes and 22 barcodes, which were revealed earlier in the ITS2 secondary structure of *Chloroidium* members [9]. The sistership of *C. orientalis* and *C. engadinensis* was supported by a number of unique CBCs/hCBCs in the barcode alignment (Figure 5): at positions 19 (A-U pair (1)) and 20 (C-G pair (3)) at the base of Helix I, and position 44 (U•G pair (6)) in Helix III. An hCBC (G•U/G-C) located in the basal base pair of helix III (position 33) differentiated *C. orientalis* from *C. engadinensis*. Two ribotypes of *C. orientalis* differed in position 38 (U-A pair (2) vs. unpaired U C bases (8)).

## 3. Discussion

We isolated three strains of *Chlorella*-like coccoid green alga and examined them using an integrative approach including light and confocal microscopy and molecular phylogenetics. The results of the 18S rDNA sequence comparisons (Table S1) revealed affinity of these strains to the genus *Chloroidium*. Using molecular characteristics (phylogenetic reconstructions and secondary structure models) and phenotypic features, we found evidence to describe our strains as a new species, *Chloroidium orientalis*. The new species is characterized by a relatively long branch in the phylogenetic tree (Figure 3), reflecting a large number of substitutions differentiating it from the rest of the genus, as well as elevated *p*-distances (see Results). Most of these molecular characters were contributed by the divergent ITS rDNA sequences (Table 1).

Phylogenetic analyses resolved *C. orientalis* as a sister of *C. engadinensis* in a clade comprising five species out of 10 genus members (Figure 3). Close affinity between *C. orientalis* and *C. engadinensis* was supported by not only significance thresholds, but also a number of shared marker substitutions in the conserved domains of ITS2 (Figure 4). Despite these synapomorphic characters, unique marker CBCs and hCBCs differentiated *C. orientalis* from *C. engadinensis*, as well as two ribotypes of *C. orientalis* from each other (Figure 5).

*C. orientalis* is characterized by ellipsoid to spherical cells, the presence of unequally sized autospores, and parietal band-shaped to cup-shaped chloroplasts with a pyrenoid (Figure 1 and Figure 2), and these features correspond to the diagnosis of the genus *Chloroidium*. Although the diagnosis stresses that the cup-shaped chloroplast is not typical for the genus representatives, at least two species (*C. lobatum* and *C. laureanum*, former *Parachloroidium* members) have chloroplasts of such type [14]. The set of morphological characters described in *C. orientalis* does not fit diagnoses of any known species. Although most *Chloroidium* taxa have ellipsoid cells, species with spherical cells are also members of the genus [9]. In this respect, the new species is intermediate between those characterized exclusively by ellipsoid cell shape and former members of the genus *Parachloroidium* with spherical cells. Unlobed chloroplasts (Figure 1) are not typical for *Chloroidium*, and this feature sets *C. orientalis* apart from the rest of the genus. In most species, the chloroplasts are deeply lobed (*C. ellipsoideum*, *C. lichenum, C. antarcticum, C. lobatum*, and *C. laureanum*) or have a wavy margin (*C. arboriculum, C. saccharophilum, C. engadinensis,* and *C. viscosum*). Another feature of *C. orientalis* that is rare in the genus is a distinct pyrenoid with starch grains. Similar pyrenoids were described in *C. lichenum* and *C. ellipsoideum*, which are members of the same clade (Figure 3), but these species differ from *C. orientalis* in chloroplast morphology.

The most striking difference between *C. orientalis* and other species of *Chloroidium* is the mode of reproduction. Like other taxa in the Watanabeales, the genus members reproduce through unequally sized autospores in small and large autosporangia [9]. In addition to that *C. arboriculum* and *C. viscosum* produce small autospores of equal size [8,9]. However, only *C. orientalis* produces three types of autospores: autospores of equal small size, autospores of equal large size, and autospores of unequal size (one large broadly ellipsoidal and 3–7 small narrowly ellipsoidal) (Figure 1 and Figure 2). Even a greater diversity of autospore types was described in *C. antarcticum*, which has autospores of equal small size with narrowly ellipsoidal shape and three types of autospores of unequal size: (1) in autosporangia with 4–8 spores containing large and small autospores of the same broadly ellipsoidal cell-shape; (2) in sporangia with 4–16 cells containing one large broadly ellipsoidal cell and other small narrowly ellipsoidal cells, and (3) in sporangia with two large and two small broadly ellipsoidal cells. It was hypothesized that variability and flexibility in autospore formation may be advantageous under fluctuating environmental conditions that are typical for non-aquatic habitats where *Chloroidium* occurs [9].

## 4. Materials and Methods

### 4.1. Strain Origin, Culture Conditions and Light Microscopy

Three strains of coccoid green alga were isolated from soil samples and concrete wall biofouling collected in the temperate monsoon climate zone in the Primorsky Territory, Russia (one sample) and in Jilin Province, China (two samples). Sampling was carried out using standard methods [15]. The strains were isolated using the micro-pipette method [16] and cultured in liquid nutrient medium Waris-H [17] at 20–22 °C with a photon fluence 17.9–21.4 μmol photons·m^−2^ s^−1^ in a 16:8 h light:dark cycle. The strains were maintained in the culture collection of the Laboratory of Botany in the Federal Scientific Center of East Asian Terrestrial Biodiversity, Russian Federation (strains number VCA-45 (Се2Е), VCA-46 (Chb1) and VCA-47 (Chb2)) and their dried biomass was deposited to the Herbarium of the Federal Scientific Center of East Asian Terrestrial Biodiversity, Russia (exsiccatum numbers VLA-CA-0963, VLA-CA-1108, VLA-CA-1109).

The morphology of vegetative and reproductive cells was examined using an Olympus BX 53 light microscope equipped with Nomarski DIC optics and Olympus DP27 digital camera. Cultures were repeatedly examined throughout the lifecycle stages, i.e., in cultures of different ages since transfer.

For confocal microscopy algae were fixed in FAA (3.7%: formaldehyde: 50% ethanol: 5% acetic acid) for 20 min, then rinsed twice and counterstained with DAPI (4,6-diamidino-2-phenylindole, Molecular Probes Inc., Eugene, OR, USA) at the final concentration of 5 µg/mL. After another rinse of samples, the fluorescence was detected with LSM 710 LIVE confocal laser scanning microscope (Carl Zeiss, Germany) at the Instrumental Centre of Biotechnology and Gene Engineering of FSCEATB FEB RAS. DAPI fluorescence detected at 410–497 nm and autofluorescence of chloroplasts was recorded in the additional emission channel after 600 nm using Plan-Apochromat 63x/1.40 Oil DIC M27 objective with digital zoom. 3D files of the captured images were recorded and analyzed with ZEN microscope software.

### 4.2. DNA Extraction, Amplification and Sequencing

Cultures were harvested during exponential growth phase and concentrated by centrifugation. Total genomic DNA was extracted as described previously by Echt et al. [18] with some modifications [19]. SSU rDNA and the internal transcribed spacer (ITS) region of rDNA were amplified using 82F (5′-GAAACTGCGAATGGCTC-3′; [20]) and ITS4R (5′-TCCTCCGCTTATTGATATGC-3′; [21]) primers and sequenced with primers 82F, 528F (5′-CGGTAATTCCAGCTCC-3′; [22]), 920F (5′-GAAACTTAAAKGAATTG-3′; [23]), Bd18SF1 (5′-TTTGTACACACCGCCCGTCGC-3′; [24]) and ITS4R. PCR amplification was performed using the Encyclo Plus PCR kit (Evrogen, Moscow, Russia) with a T100 Thermal Cycler (Bio-Rad Laboratories, Inc., Hercules, CA, USA) and the following parameters 95 °C, 5 min; 38 × (95 °C, 20 s; 55 °C, 20 s; 72 °C, 2 min 40 s); 72 °C, 5 min. The PCR products were purified by ExoSAP-IT PCR Product Cleanup Reagent (Affymetrix Inc., Santa Calra, CA, USA) and sequenced in both directions using an ABI 3500 genetic analyzer (Applied Biosystems, Rockville, MD, USA) with a BigDye terminator v.3.1 sequencing kit (Applied Biosystems, MD, USA). Sequences were assembled with the Staden Package v.1.4 [25], aligned manually in the SeaView program [26]. Sequences were deposited in GenBank under accession numbers MZ558750–MZ558752 (Table S2).

### 4.3. Phylogenetic Analysis

Maximum likelihood (ML) analysis was carried out using PAUP 4.0b10 [27]. Bayesian inference (BI) was performed using MrBayes 3.1.2 [28]. To determine the most appropriate DNA substitution model for our datasets, the Akaike information criterion (AIC; [29]) was applied with jModelTest 2.1.1 [30]. MEGA v.7.0.26 [31] was used to estimate interspecific pairwise distances (*p*-distances). ML analysis was done using heuristic searches with a branch-swapping algorithm (tree bisection-reconnection). In BI, four runs of four Markov chains were carried out for 2 million generations, sampling every 100 generations for a total of 20,000 samples. Convergence of the two chains was assessed, and stationarity was determined according to the ‘sump’ plot with the first 5000 samples (25%) discarded as burn-in; posterior probabilities were calculated from trees sampled during the stationary phase. The robustness of the ML trees was estimated by bootstrap percentages (BP; [32]) and posterior probabilities (PP) in BI. BP < 50% and PP < 0.95 were not considered. ML-based bootstrap analysis was inferred using the web service RAxML version 7.7.1 (http://embnet.vital-it.ch/raxml-bb/; accessed on 15 July 2021; [33]).

The Mfold web server (http://www.unafold.org/mfold/applications/rna-folding-form.php; accessed on 20 July 2021; [34]) was used with the default settings to generate the folding pattern of ITS2 ribosomal RNA secondary structure. An ITS2 model was constructed based on models proposed by Darienko et al. [9]. For the ITS2/CBC approach, the conserved regions of ITS2 were extracted and analyzed following Darienko et al. [9].

## Figures and Tables

**Table 1:** *P*-distances among *Chloroidium* species based on the aligned ITS (ITS1–5.8S–ITS2) region (730 positions). Standard error estimates are shown above the diagonal.

№	Species	1	2	3	4	5	6	7	8	9	10
1	*C. saccharophilum*		0.0166	0.0157	0.0139	0.0147	0.0127	0.0135	0.0149	0.0130	0.0165
2	*C. laureanum*	0.2402		0.0162	0.0174	0.0168	0.0174	0.0191	0.0176	0.0165	0.0154
3	*C. lobatum*	0.2443	0.2520		0.0173	0.0175	0.0165	0.0166	0.0162	0.0159	0.0181
4	*C. viscosum*	0.1573	0.2555	0.2707		0.0148	0.9621	0.0112	0.0133	0.0132	0.0165
5	*C. antarcticum*	0.1859	0.2778	0.2953	0.1725		0.0145	0.0142	0.0175	0.0167	0.0154
6	*C. engadinensis*	0.1494	0.2639	0.2771	*0.0834*	0.1612		0.0118	0.0139	0.0135	0.0160
7	*C. orientalis*	0.1797	0.2666	0.2927	0.1277	0.1879	**0.1153**		0.0146	0.0138	0.0159
8	*C. ellipsoideum*	0.1630	0.2274	0.2703	0.1376	0.1751	0.1469	0.1582		0.0067	0.0169
9	*C. lichenum*	0.1490	0.2250	0.2694	0.1387	0.1786	0.1443	0.1418	*0.0505*		0.0166
10	*C. arboriculum*	0.2434	0.2234	0.2601	0.2718	0.2806	0.2720	0.2694	0.2617	0.2450	

## Data Availability

The data presented in this study are available on request from the corresponding author. In addition, the data that support the findings of this study are openly available in GenBank (see Table S2).

## Supplementary Materials

The following are available online at www.mdpi.com/article/10.3390/plants10122560/s1, Table S1: P-distances among *Chloroidium* species based on the aligned 18S region (1187 positions). Standard error estimates are shown above the diagonal. Table S2: Taxon names, strain information, locality, EMBL/GenBank accession numbers for the taxa used in our analyses. Sequences obtained in this study are boldfaced.
